# Resistance Training and Respiratory and Immune‐Related Outcomes in Older Adults: A Randomized Controlled Trial

**DOI:** 10.1155/jare/6364341

**Published:** 2026-11-02

**Authors:** Anamei Silva-Reis, Rômulo Antonio Costa de Oliveira, Meiry Souza Moura-Maia, Eduarda Teodora Rachid-Wolpp, Naiara Nadia Moreira-Silva, Luis Vicente Franco de Oliveira, Andre Luis Lacerda Bachi, Vicente Aprigliano, Omid Salehian, Regiane Albertini, Guilherme Eustáquio Furtado, Alberto Souza Sá Filho, Daiana Stolz, Rodolfo P. Vieira

**Affiliations:** ^1^ Laboratory of Pulmonary and Exercise Immunology (LABPEI) Evangelical University of Goias (UniEVANGELICA) Anápolis Goiás 75083-515 Brazil; ^2^ Laboratory of Pulmonary Rehabilitation Evangelical University of Goias (UniEVANGELICA) Anápolis Goiás 75083-515 Brazil; ^3^ Postgraduate Program in Health Science Santo Amaro University (UNISA) São Paulo São Paulo 04829-300 Brazil; ^4^ Department of Otorhinolaryngology – Head and Neck Surgery Federal University of Sao Paulo (UNIFESP) São Paulo São Paulo 04038-032 Brazil unifesp.br; ^5^ Escuela de Ingeniería de Construcción y Transporte Pontificia Universidad Católica de Valparaíso Avda Brasil 2147 Valparaíso 2362804 Chile ucv.cl; ^6^ Department of Sport Nutrition and Fitness Applied and Science University Tehran 13114-16846 Iran; ^7^ Laboratory of Pulmonary and Exercise Immunology (LABPEI) Federal University of Sao Paulo (UNIFESP) São José Dos Campos Goiás 12231-280 Brazil unifesp.br; ^8^ SPRINT-Sport Physical Activity and Health Research & Innovation Center Polytechnic Institute of Coimbra Coimbra 3045-093 Portugal ipc.pt; ^9^ Clinic of Pneumology Medical Center-University of Freiburg Freiburg 79106 Germany uniklinik-freiburg.de; ^10^ Brazilian Institute of Teaching and Research (IBEP) Anápolis Goiás 75064-420 Brazil

**Keywords:** cytokines, inflammation, older adults, resistance training, respiratory muscle strength

## Abstract

Age‐related declines in pulmonary function and immune regulation increase morbidity and mortality in older adults. Physical activity mitigates these effects, but the isolated impact of resistance training (RT) on lung function and immune responses remains unclear. Sixty‐nine older adults without participation for at least one year in structured exercise programs (aged 60–85 years) were recruited from a community extension program at a Brazilian university and randomized into an RT group (*n* = 31) or a control group (*n* = 38). This randomized controlled trial evaluated the effects of a 12‐week supervised RT program performed three times per week. Primary outcomes included spirometry parameters (FVC, FEV_1_, FEV_1_/FVC, PEF, and FEF_25_–_75_%), respiratory muscle strength (MIP and MEP), and inflammatory biomarkers (IL‐6, IL‐10, TNF‐α, and Klotho) in plasma and exhaled breath condensate. Secondary outcomes included handgrip strength, sit‐to‐stand performance, oxygen saturation, and body composition. Normally distributed outcomes were analyzed using repeated‐measures ANOVA, whereas non‐normally distributed longitudinal outcomes were analyzed using the Brunner–Langer approach for factorial longitudinal data. Between‐group differences in pre‐to‐post changes were additionally estimated using bootstrap 95% confidence intervals. No significant Group × Time interactions were observed for spirometric parameters. Significant Group × Time interactions were observed for MIP and MEP; however, significant pre‐to‐post changes were observed in the control group but not in the RT group. A significant Group × Time interaction was also observed for sit‐to‐stand performance, with improvement in the control group but not in the RT group. No significant Group × Time interactions were observed for handgrip strength or oxygen saturation. Significant Group × Time interactions were observed for plasma IL‐6, IL‐10, and Klotho (all *p* < 0.001), as well as exhaled breath condensate IL‐6 (*p* = 0.006) and Klotho (*p* = 0.003). In contrast, no significant interactions were observed for plasma TNF‐α, exhaled breath condensate IL‐10, or exhaled breath condensate TNF‐α. Twelve weeks of supervised RT were associated with differential changes in respiratory muscle strength, functional capacity, and selected systemic and airway biomarkers in older adults. Significant Group × Time interactions were observed for MIP, MEP, the 1‐min sit‐to‐stand test, and selected inflammatory and aging‐related biomarkers, but not for pulmonary function. These findings indicate that the effects of RT varied across outcomes, rather than demonstrating consistent improvement across all assessed measures.

**Trial Registration:** ClinicalTrials.gov identifier: NCT06682221

**Highlights and Key Points**
• A 12‐week resistance training program improved respiratory muscle strength and physical performance in previously untrained older adults.
• Resistance training reduced systemic and pulmonary pro‐inflammatory cytokines while increasing anti‐inflammatory and anti‐aging biomarkers.

## 1. Introduction

In older adults, immunosenescence alters pulmonary and systemic immunity, affecting innate and adaptive responses and cytokine regulation, which contributes to the development of inflammaging [1, 2]. Inflammaging is characterized by reduced number of leukocytes, including reduced proliferation and activation, loss of receptors, and mitochondrial working, impairing pathogen defense [1–3]. Strategies, such as vaccination, immunomodulation, and anti‐inflammatory therapies, may reduce this burden [4–6]. Inflammaging is related to reduced muscle strength [7], lean mass [7], cardiovascular [8] and respiratory function [9], and increasing dependency in daily activities [10]. These age‐related alterations may negatively affect ventilatory performance, exercise tolerance, and overall physical functioning, even in the absence of diagnosed pulmonary disease [11]. Respiratory muscle dysfunction can cause insufficiency, hyperventilation, and reduced exercise tolerance, further raising morbidity and mortality and negatively impacting health and quality of life [12, 13].

Physical inactivity, highly prevalent among older adults, is a major risk factor for global mortality, accounting for 6% of deaths [14–16]. It is linked to reduced forced expiratory volume (FEV1) and forced vital capacity (FVC), as well as lower respiratory muscle strength and impaired physical performance [17, 18]. Aging also alters the immune system, often creating a chronic pro‐inflammatory state. Elevated interleukin‐6 (IL‐6) and tumor necrosis factor‐alpha (TNF‐α) increase disability, mortality, and risks of type II diabetes, cardiovascular and neurodegenerative diseases, and cancer, while interleukin‐10 (IL‐10) regulation may help predict inflammaging progression [19–21]. Because respiratory muscles are metabolically active skeletal muscles, they may be susceptible to the systemic effects of chronic inflammation and age‐related neuromuscular decline, potentially influencing ventilatory efficiency and respiratory performance during physical exertion [21–23]. In addition, older adults with stage 1 hypertension, such as those included in the present study, may present a higher burden of systemic inflammatory activity and endothelial dysfunction, which can compromise respiratory and functional capacity [1, 24].

While regular physical activity and combined exercise modalities have been shown to benefit immune function [25] and lung health [26] in older adults, the isolated effects of resistance training (RT) on pulmonary function, respiratory muscle strength, and systemic and pulmonary inflammatory profiles remain poorly understood in older adults without recent participation in structured exercise program. RT is recognized as an important strategy to improve skeletal muscle strength, neuromuscular performance, and functional capacity. These systemic adaptations may also contribute to improved ventilatory efficiency through enhanced respiratory muscle performance and reduced ventilatory demand during exercise [27]. Moreover, most studies investigating immune adaptations to exercise have focused exclusively on systemic biomarkers, with very few evaluating inflammation directly at the airway level [28, 29]. However, airway‐specific inflammatory assessment may provide additional insights into local pulmonary responses that are not captured by systemic markers alone, particularly in aging populations exposed to chronic inflammatory burden [30]. This gap is particularly relevant in populations with elevated cardiometabolic burden, such as older adults without participation for at least 1 year in structured exercise programs with hypertension, who may present greater inflammatory dysregulation and impaired respiratory responses [31].

In particular, the use of exhaled breath condensate (EBC), a noninvasive method capable of capturing airway‐specific cytokines and oxidative markers, remains extremely limited in exercise‐based interventions [28, 29]. To the best of our knowledge, no previous study has examined EBC‐derived pulmonary inflammation/immune response in older adults without participation for at least 1 year in structured exercise programs undergoing RT. Although the relationship between RT and airway inflammatory biomarkers is not yet fully established, investigating these markers may contribute to a better understanding of the integrative physiological adaptations associated with exercise in aging populations. Additionally, pulmonary inflammatory processes may contribute to ventilatory limitations and reduced exercise tolerance [32], reinforcing the relevance of evaluating pulmonary biomarkers alongside functional and respiratory outcomes.

We hypothesized that a 12‐week RT program would improve pulmonary function (FEV1, FEV1/FVC, PEF, and FEF25%–75%), respiratory muscle strength (MIP and MEP), functional capacity (1‐min sit‐to‐stand), and handgrip strength in the study population. We also expected RT to be associated with changes in systemic and pulmonary inflammatory markers, including reduced IL‐6 and TNF‐α and increased IL‐10 and Klotho concentrations. Because oxygen saturation remains typically stable in individuals without diagnosed pulmonary disease, even under physical stress, peripheral oxygen saturation was included as a physiological response variable rather than an indicator of respiratory pathology or disease severity [33]. Therefore, any observed changes in oxygen saturation should be interpreted within a functional physiological context rather than as clinically relevant desaturation events.

Therefore, this study aims to investigate whether RT can significantly improve pulmonary function and immune response in a group within the study population. Given that respiratory muscle weakness, impaired ventilatory efficiency, and airway inflammation directly contribute to limitations in functional performance, examining these physiological domains together helps clarify the mechanisms through which RT may enhance physical functioning in daily life. Furthermore, the combined assessment of systemic and airway‐specific biomarkers may provide novel insights into the interaction between RT, respiratory health, and inflammatory regulation in older adults.

## 2. Materials and Methods

### 2.1. Study Design, Ethical Approval, and Participants

This randomized, controlled, parallel‐group trial evaluated the effects of RT on pulmonary function, respiratory muscle strength, functional capacity, and systemic and pulmonary inflammatory responses in older adults without recent participation in structured exercise programs. The study followed CONSORT guidelines to ensure methodological rigor and transparency (Figure 1).

**FIGURE 1:**
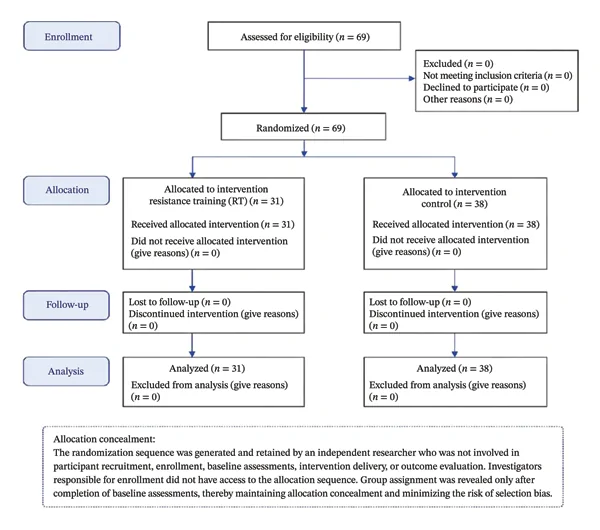
CONSORT 2025 flow diagram of participant recruitment, randomization, follow‐up, and analysis. CONSORT 2010 flow diagram of participant recruitment, allocation, follow‐up, and analysis. A total of 69 participants were assessed for eligibility and randomized to either the resistance training (RT; *n* = 31) or control (CON; *n* = 38) group. All randomized participants received their allocated intervention, completed follow‐up, and were included in the final analysis. RT, resistance training.

Participants were randomly allocated to an RT intervention group, performing progressive RT three times per week for 12 weeks, or a control group maintaining usual daily activities, with assessments conducted at baseline and postintervention. Ethical approval dated June 1, 2023, was granted by the Ethics Committee of the Evangelical University of Goiás (protocol no. 6.095.284). The study was registered on the Brazil Platform (68189923.5.0000.5076). The study record and protocol‐related information are publicly available through ClinicalTrials.gov; however, a separate statistical analysis plan is not publicly available. Individual participant data, statistical code, and other study materials are not publicly available but may be made available upon reasonable request to the corresponding author, subject to applicable ethical and data protection requirements.

Sixty‐nine volunteers were recruited from the UniEVANGÉLICA social project and social media; after providing informed consent, 31 were assigned to RT and 38 to control. Inclusion criteria included age 60–85 years, ability to complete assessments, and attendance of ≥ 75% of RT sessions. Physical activity status was assessed during anamnesis. To be eligible, participants could not have participated in structured exercise programs for at least 1 year. This criterion was adopted to minimize potential influences of recent exercise training on baseline physiological and inflammatory outcomes. Exclusion criteria included smoking, alcohol use disorder, or neurological conditions. Participants allocated to the control group were instructed to maintain their usual daily activities and not to engage in structured physical activity, including RT, throughout the 12‐week period. Based on baseline systolic and diastolic values, both groups met criteria for Stage 1 hypertension according to the AHA/ACC guidelines (SBP 130–139 mmHg and/or DBP 80–89 mmHg) [34].

During baseline anamnesis, information regarding smoking history (current and former smoking status), diabetes mellitus, systemic hypertension, hypercholesterolemia, and other self‐reported comorbidities was collected. Current smokers were excluded from participation, whereas former smokers who had discontinued smoking at least 1 year before enrollment were considered eligible. Information on medication use was also collected during the clinical interview; however, medication classes were not systematically categorized for quantitative analysis. These baseline clinical characteristics are presented in Table 1. No important changes were made to the intervention protocol, outcomes, or analyses reported in the present study after trial commencement.

**TABLE 1:** Clinical and laboratory baseline characteristics of older adults.

Variable	Baseline CON	95% CI	Baseline RT	95% CI
Age (years)	69.4 ± 6.2	(66.2–72.5)	67.6 ± 6.1	(64.5–70.6)
Height (m)	1.59 ± 0.09	(1.54–1.64)	1.57 ± 0.07	(1.53–1.61)
Weight (kg)	73.4 ± 15.2	(65.71–81.09)	68.7 ± 10.8	(63.2–74.1)
BMI (kg/m^2^)	29.1 ± 5.3	(26.4–31.7)	27.6 ± 3.5	(25.8–29.3)
Fat‐free mass (kg)	25.2 ± 4.9	(22.7–27.6)	25.2 ± 4.0	(23.1–27.2)
Fat mass (%)	38.8 ± 9.9	(33.7–43.8)	37.7 ± 8.4	(33.4–41.9)
SBP (mmHg)	135.4 ± 20.1	(125.2–145.5)	131.1 ± 16.1	(122.9–139.2)
DBP (mmHg)	82.9 ± 12.7	(76.4–89.3)	81.1 ± 9.2	(76.4–85.7)
RPP	9389.2 ± 1999.9	(8377–10401)	8852.1 ± 1497.6	(8094–9609)
PP (mmHg)	52.5 ± 12.3	(46.2–58.7)	50.0 ± 13.8	(43.2–56.9)
HR (bpm)	69.1 ± 11.9	(63.0–75.1)	70.4 ± 9.9	(65.3–75.4)
Diabetes	7 from 38	N/A	4 from 31	N/A
Total cholesterol	11 from 38	N/A	4 from 31	N/A
Systemic hypertension	27 from 38	N/A	14 from 31	N/A
Current smoker	0 from 38	N/A	0 from 31	N/A
Former smoker	10 from 38	N/A	4 from 31	N/A

*Note:* RT = resistance training group; CON = control group; m = meters; Kg = kilogram; % = percentage; mmHg = millimeters of mercury. PP = pulse pressure; DBP = diastolic blood pressure; SBP = systolic blood pressure.

Abbreviations: bpm = beats per minute, FFM = fat‐free mass, N/A = nonapplicable, RPP = rate‐pressure product.

### 2.2. Sample Size

Sample size was calculated a priori using G∗Power (Version 3.1.9.4) based on a repeated‐measures ANOVA with a within‐between interaction [35]. The effect size (*f* = 0.25) was selected a priori based on Cohen’s conventional classification of a moderate effect for repeated‐measures ANOVA and was considered consistent with effect magnitudes reported in meta‐analyses of RT in older adults, which show small‐to‐moderate standardized effects on functional and strength outcomes [36, 37], *α* = 0.05, and 95% power (1−β). The model considered two groups (intervention and control) and two time points (pre‐ and postintervention), assuming a moderate correlation among repeated measures (*r* = 0.5) and no violation of sphericity was assumed (*ε* = 1). This calculation indicated that a minimum sample of 54 participants was required. To account for potential dropouts, recruitment was increased by approximately 25%, resulting in 69 enrolled participants.

### 2.3. Randomization and Blinding

Participants were randomly assigned to the intervention (RT) or control group using a computer‐generated sequence, without stratification or block constraints. The randomization sequence was generated, retained, and implemented by an independent researcher who was not involved in participant recruitment, enrollment, baseline assessments, intervention delivery, or outcome evaluation. Investigators responsible for participant enrollment did not have access to the allocation sequence. Group assignment was disclosed only after completion of all baseline assessments, thereby maintaining allocation concealment and minimizing the risk of selection bias. This simple randomization approach supported an unbiased distribution of participants and preserved internal validity.

While blinding of participants and exercise supervisors was not feasible due to the nature of the intervention, outcome assessors remained blinded to group allocation during postintervention evaluations, thereby reducing the risk of detection bias and strengthening methodological rigor.

### 2.4. Resistance Exercise Training (RT)

The RT protocol comprised seven exercises: high pull, leg extension, leg curl, biceps curl, triceps pushdown with bar, lateral raise, and machine bench press. Sessions lasted approximately 60 min and were performed three times per week for 12 weeks. All RT sessions were supervised throughout the intervention period, with adherence to the prescribed training protocol and exercise execution monitored during each session. The first week focused on exercise familiarization to ensure proper technique before progression. Each exercise was executed in three sets of 8–12 repetitions, with 1–2 min of rest between sets. Loads were progressively increased by 2%–10% per week when participants successfully completed the prescribed repetitions with full range of motion in two consecutive sessions. Training intensity was monitored through observable fatigue indicators, including concentric failure, decreased movement speed, breath holding, or prolonged isometric contractions. Assessments were conducted at baseline and 24 h after the final session to evaluate the effects of RT on physical and functional parameters [38].

To detail the training intensity, all participants underwent a one‐repetition maximum (1RM) test for each exercise before the intervention, following a validated procedure described in previous literature [39]. Training loads were prescribed as a percentage of these values, with participants beginning the program at approximately 65% of their individual 1RM and progressing toward intensities close to 80% of 1RM as adaptations occurred.

The structure of the program followed international RT recommendations for older adults [38, 40], with controlled execution emphasizing movement quality. The concentric phase of each repetition lasted about 2 s, while the eccentric phase lasted about 4 s, ensuring sufficient time under tension. Full joint range of motion was encouraged whenever feasible, respecting anatomical or functional limitations of each participant.

To minimize exercise‐order bias and distribute fatigue evenly across muscle groups, the sequence of exercises was rotated systematically across sessions, so that each exercise served as the starting point at different times throughout the 12‐week period. This organizational strategy has been recommended to reduce cumulative fatigue effects during multi‐exercise RT protocols [40]. Harms were not systematically assessed; however, participants were closely monitored throughout the intervention, and no adverse events or harms were observed.

### 2.5. Outcomes

Primary outcomes included pulmonary function (FEV1, FEV1/FVC, PEF, and FEF25%–75%), respiratory muscle strength (MIP and MEP), and systemic and pulmonary inflammatory biomarkers (IL‐6, TNF‐α, IL‐10, and Klotho). Secondary outcomes included body composition, functional performance, oxygen saturation responses during exercise, handgrip strength, and cardiovascular parameters. Detailed descriptions of all assessments are provided below. The assessments were conducted at baseline and after the three‐month intervention period.

### 2.6. Primary Outcome

#### 2.6.1. Lung Function Measurements

Participants’ lung function was evaluated using a KoKo spirometer (nSpire Health, CO, USA) following the forced maneuver protocol recommended by the Brazilian Society of Pneumology and Tisiology, with reference values for the Brazilian population [41]. Spirometry was performed following standardized procedures, including administration of a rapid‐acting bronchodilator (Salbutamol sulfate 400 μg) in accordance with national clinical recommendations. However, only prebronchodilator values were used for statistical analyses, and postbronchodilator measurements were collected solely for clinical standardization purposes, and to investigate possible undiagnosed respiratory abnormalities. Bronchodilator responsiveness was not evaluated, and no pre‐versus‐postbronchodilator comparisons were performed. In addition, no participant demonstrated clinically significant bronchodilator responsiveness.

Analyzed parameters included FVC, FEV1, FEV1/FVC ratio, PEF, and FEF25%–75%. These variables were analyzed to investigate the effects of RT on pulmonary function in older adults without recent participation in structured exercise programs. These measures provide a comprehensive assessment of pulmonary function, enabling detection of respiratory impairments according to standardized spirometry recommendations [41, 42]. Only spirometry tests that met the American Thoracic Society/European Respiratory Society (ATS/ERS) acceptability and repeatability criteria were included in the analyses. Participants who were unable to perform acceptable spirometric maneuvers were excluded only from the spirometry analyses but remained included in the analyses of the other study outcomes [42, 43].

#### 2.6.2. Respiratory Muscle Strength Measurements

Maximal inspiratory (MIP) and expiratory pressures (MEP) were measured using a manual manovacuometer, a validated method for assessing respiratory muscle strength [43, 44]. Participants remained seated and used a nose clip during all maneuvers to prevent nasal air leakage.

For MIP assessment, participants performed a maximal inspiratory effort after full expiration, whereas for MEP assessment, participants performed a maximal expiratory effort after full inspiration. Participants performed at least three maximal inspiratory and expiratory maneuvers according to standardized laboratory procedures. Maneuvers were considered acceptable when performed without air leakage and with sustained maximal effort, and the highest reproducible value (defined as two maneuvers within 10% variability) was used for analysis [43, 44]. Pressure values were recorded in cmH_2_O.

Strong verbal encouragement was provided throughout testing to ensure maximal effort. This test provides important information on respiratory muscle function and allows evaluation of the effectiveness of interventions aimed at improving respiratory performance.

Absolute MIP and MEP values (cmH_2_O) were used for statistical analyses because the primary objective was to investigate changes in respiratory muscle performance following RT within and between groups [29], rather than to establish clinical classification based on predicted values.

#### 2.6.3. Analysis of Pulmonary Inflammatory and Fibrotic Biomarkers

Pulmonary inflammatory and fibrotic biomarkers were assessed using EBC collected with an RT‐Tube (Respiratory Research, TX, USA), following the manufacturer’s guidelines and established methodological recommendations for EBC collection and processing [28, 45, 46]. Participants breathed at tidal volume for 15 min, and samples were subsequently stored at −86°C until analysis. Collection procedures were conducted according to standardized methodologies to improve sample reproducibility and minimize contamination during EBC acquisition [46].

The biomarkers analyzed included IL‐6 (DY206), IL‐10 (DY217B), TNF‐α (DY210), and Klotho (DY5334). Concentrations were determined using commercially available DuoSet ELISA kits (R&D Systems, MN, USA). The analytical performance of ELISA‐based assays for inflammatory mediators in EBC has previously been evaluated, including at the low concentrations characteristic of this matrix [47].

Klotho has previously been detected and quantified in human EBC or breath condensate using ELISA‐based methods in older adults [48], individuals with asthma undergoing exercise training [28], women with overweight or obesity undergoing exercise [29], older adults undergoing high‐intensity RT [49], and older adults receiving whey‐protein supplementation [50]. In the present study, Klotho was quantified using the R&D Systems DuoSet Klotho assay (DY5334), according to the manufacturer’s instructions and standardized laboratory procedures. However, formal analytical validation and reliability data specifically for Klotho measurement in the EBC matrix remain limited.

#### 2.6.4. Analysis of Systemic Inflammatory and Fibrotic Biomarkers

A total of 5 mL of venous blood was collected from each participant using a sterile vacuum tube containing EDTA K3 as an anticoagulant. Immediately after collection, the blood was centrifuged at 1000 g for 7 min at a temperature of 4°C. After centrifugation, the plasma was carefully separated and stored at −86°C until cytokine assays for IL‐6, IL‐10, TNF‐α, and Klotho protein were conducted.

These biomarkers were measured using DuoSet ELISA kits (R&D Systems), selected due to their analytical sensitivity and widespread use in cytokine quantification protocols. Plasma concentration readings were obtained using the Spectramax I3 microplate reader (Molecular Devices, CA, USA), according to the manufacturer’s instructions and standardized laboratory procedures for cytokine quantification [28, 45]. All procedures followed standardized laboratory protocols for plasma biomarker analysis.

### 2.7. Secondary Outcomes

#### 2.7.1. Anthropometric and Body Composition Assessment

Anthropometric data, including age, body weight, height, and body mass index (BMI), were collected to allow a comprehensive evaluation of body mass distribution and potential health risks associated with body composition. Participants’ body composition was assessed using bioimpedance analysis (BIA; Bioscan 920 II‐S, Maltron, UK), which estimates lean mass and body fat percentage [28] based on electrical conductivity properties.

For the BIA measurement, participants were positioned in the supine position on a nonconductive surface, with arms slightly abducted from the trunk and legs separated to avoid contact between body segments. The skin was cleaned and dried before electrode placement. Disposable electrodes were placed according to the manufacturer’s tetrapolar configuration, preferably on the right side of the body: Two electrodes were positioned on the hand/wrist region and two on the foot/ankle region. The hand electrodes were placed over the metacarpal and carpal regions, whereas the foot electrodes were placed over the metatarsal and talus/ankle regions. Electrode adhesion and cable connections were checked before each test, and participants were instructed to remain still and relaxed during the measurement. The device automatically estimated body composition parameters, including fat‐free mass and fat mass percentage, which were recorded for analysis [28].

#### 2.7.2. Functional Capacity and Oxygen Desaturation

Lower‐limb strength and endurance were assessed using the 1‐min sit‐to‐stand test (1‐MSTST) at baseline and after the 12‐week intervention [51, 52]. Before testing, participants remained seated at rest for at least 10 min. The test was performed using a standardized chair positioned on a stable surface. Participants were instructed to complete as many sit‐to‐stand cycles as possible in 60 s without using their arms for assistance. Standardized verbal instructions were provided before each test by a trained evaluator. The total number of completed repetitions was recorded for analysis. SpO_2_ was monitored before, during, and immediately after the test using the same digital fingertip oximeter (Oxy Control, Geratherm, Germany). The change in SpO_2_ was calculated by comparing pre‐ and post‐test values.

#### 2.7.3. Handgrip Strength

Handgrip strength was measured using a calibrated digital hand dynamometer (JAMAR), a validated instrument for assessing isometric grip force and strongly recommended for screening sarcopenia and overall muscle function in older adults [45]. Participants received standardized instructions and a demonstration from a trained evaluator. Tests were performed with the participant seated on a standard chair, feet flat on the floor, hips and knees at 90°, and the dominant arm positioned with the elbow flexed at 90°, wrist neutral, and forearm unsupported. Three maximal contractions of 3–5 s were performed, with 60‐s intervals between trials. The highest value was recorded for analysis [45].

#### 2.7.4. Missing Data

No participants were lost to follow‐up or withdrew from the study; therefore, no imputation procedures were required.

### 2.8. Statistical Analysis

Statistical analyses were performed using IBM SPSS Statistics Version 27.0 (IBM Corp., Armonk, NY, USA). The Brunner–Langer longitudinal analyses and bootstrap estimation of 95% confidence intervals were performed using R Version 4.4.3 (R Foundation for Statistical Computing, Vienna, Austria) with the nparLD package Version 2.2. Graphical presentations were generated using GraphPad Prism Version 8.0.1 (GraphPad Software, San Diego, CA, USA). Descriptive results are presented as mean ± standard deviation (SD) for normally distributed variables and as median and interquartile range (IQR) for non‐normally distributed variables. Data distribution was assessed using the Shapiro–Wilk test.

For baseline comparisons between groups, normally distributed continuous variables were analyzed using independent‐samples *t*‐tests, whereas non‐normally distributed continuous variables were analyzed using the Mann–Whitney *U* test. For pre‐to‐post intervention comparisons within each group, paired‐samples *t*‐tests were used for normally distributed variables and the Wilcoxon signed‐rank test for non‐normally distributed variables.

For repeated measurements obtained at baseline and after the intervention, the Brunner–Langer nonparametric analysis for factorial longitudinal designs was used as the primary inferential approach. This method accounts for the repeated‐measures structure and was used to evaluate the effects of Group (RT vs. CON), Time (pre vs. post), and the Group × Time interaction. The ANOVA‐type statistic (ATS) and corresponding *p*‐values were reported. When a significant Group × Time interaction was identified, simple effects were examined to characterize within‐group changes over time and between‐group differences at each time point. *p*‐values for the four simple‐effect comparisons were adjusted using the Holm procedure. Between‐group comparisons at each time point were performed using the Mann–Whitney *U* test.

To provide 95% confidence intervals for the magnitude of the intervention effects, bootstrap confidence intervals were calculated for the between‐group difference in pre‐to‐post change for all main intervention outcomes, including pulmonary function, respiratory muscle strength, functional performance, handgrip strength, oxygen saturation, and plasma and EBC biomarkers. Individual change was calculated as postintervention minus baseline, and the difference between the mean change in the RT group and the CON group (RT − CON) was estimated using 10,000 bootstrap resamples performed separately within each group. The 95% confidence intervals were obtained using the percentile method, corresponding to the 2.5th and 97.5th percentiles of the bootstrap distribution. A fixed random seed of 2026 was used to ensure reproducibility. These confidence intervals represent the between‐group difference in change in the original measurement units and are complementary to the Brunner–Langer analysis; they do not represent confidence intervals for the ATS.

Potential outliers were assessed by visual inspection of boxplots for all study variables. Potentially discrepant observations were checked against the original dataset for possible data‐entry or measurement errors. No data‐entry or measurement errors were identified, and all observations were retained in the primary analyses, including the primary Brunner–Langer longitudinal analyses. Sensitivity analyses were subsequently performed by excluding particularly extreme observations considered potentially influential. For SpO_2_, five extreme observations were excluded: one observation from the RT group at the postintervention pretest assessment and four observations from the control group, including one at baseline pretest and three at postintervention pretest. For plasma Klotho, four extreme observations were excluded: two from the control group at baseline and two from the RT group after the intervention. For the sensitivity analyses, only the specific observations considered potentially influential were excluded, while the remaining observations from the same participants were retained. The corresponding Brunner–Langer longitudinal analyses were then repeated. Thus, the primary longitudinal analyses included all available observations, whereas the exclusion of extreme observations was restricted to the sensitivity analyses. Statistical significance was set at *p* < 0.05. Statistical methods were reported in accordance with the CHAMP checklist [53].

As sensitivity analyses, adjusted postintervention models included the intervention group as the primary factor, the corresponding baseline outcome as a covariate, and hypertension, hypercholesterolemia, and smoking history as additional covariates. Conventional ANCOVA was used when the homogeneity of variance assumption was met, whereas generalized linear models with robust covariance estimates were used when heteroscedasticity was detected. Smoking history was classified as never versus former smoking because no participant was a current smoker. Given the number of outcomes and statistical comparisons, a sensitivity analysis accounting for multiplicity was performed for the eight Group × Time comparisons of pulmonary and systemic inflammatory and fibrotic biomarkers. The Holm procedure was applied to control the family‐wise error rate across these eight comparisons. For comparisons reported as *p* < 0.001, a value of 0.001 was used for the multiplicity‐adjustment calculation. The multiplicity‐adjusted analysis was considered complementary to the prespecified Brunner–Langer longitudinal analysis and did not replace the primary analysis. The additional sensitivity analyses were conducted to assess the robustness of the findings.

## 3. Results

### 3.1. Participant Characteristics and Adherence

All 69 randomized participants completed the intervention and were analyzed according to their original group allocation. Baseline demographic, anthropometric, body composition, and hemodynamic characteristics were comparable between the CON and RT groups, with no significant between‐group differences (Table 1).

Clinical, body composition, and hemodynamic variables before and after the intervention are presented in Table 2. Within the RT group, fat‐free mass, fat mass, systolic blood pressure, diastolic blood pressure, and heart rate showed significant pre‐to‐post changes, whereas no significant changes were observed for these variables in the CON group. BMI, rate‐pressure product, and pulse pressure did not show significant within‐group changes.

**TABLE 2:** Clinical and anthropometric characteristics of older adults before and after the intervention.

Variable	CON group		*p* value	RT group		*p* value
	Pre	Post		Pre	Post	
BMI (kg/m^2^)	29.1 ± 5.4	29.1 ± 5.5	*p* = 0.8541	27.6 ± 3.51	27.9 ± 3.4	*p* = 0.5404
	−0.2799 to 0.3361 (95% CI)			−0.1327 to 0.2480 (95% CI)		

FFM (kg)	25.2 ± 4.5	26.1 ± 4.7	*p* = 0.0780	25.2 ± 4.05	26.0 ± 4.2	*p* = 0.0021
	−1.728 to 0.09658 (95% CI)			−1.187 to −0.2929 (95% CI)		

Fat mass (%)	38.8 ± 9.9	37.9 ± 10.6	*p* = 0.1399	37.7 ± 8.4	37.9 ± 9.0	*p* = 0.0315
	−0.3013 to 2.054 (95% CI)			0.07277 to 1.461 (95% CI)		

SBP (mmHg)	135.4 ± 20.1	134.0 ± 20.2	*p* = 0.6905	131.1 ± 16.1	128.4 ± 17.4	*p* = 0.0370
	−5.865 to 8.760 (95% CI)			0.4677 to 13.77 (95% CI)		

DBP (mmHg)	82.9 ± 12.7	82.8 ± 9.6	*p* = 0.9630	81.1 ± 9.3	76.5 ± 9.1	*p* = 0.0049
	−3.349 to 3.507 (95% CI)			2.380 to 11.86 (95% CI)		

RPP	9389 ± 1999	9377 ± 1724	*p* = 0.5722	8852 ± 1467	8601 ± 1577	*p* = 0.5116
	−1072 to 601.7 (95% CI)			−387.5 to 761.3 (95% CI)		

PP (mmHg)	52.5 ± 12.3	51.1 ± 17.5	*p* = 0.6163	50.0 ± 13.7	51.9 ± 13.6	*p* = 0.5237
	−4.121 to 6.857 (95% CI)			−8.058 to 4.191 (95% CI)		

HR (bpm)	69.1 ± 11.9	70.2 ± 11.3	*p* = 0.6282	70.4 ± 9.9	65.2 ± 9.4	*p* = 0.0008
	−5.294 to 3.240 (95% CI)			2.455 to 8.022 (95% CI)		

*Note:* Data are expressed as mean ± standard deviation and 95% confidence intervals (95% CI); m = meters; Kg = kilogram; % = percentage; mmHg = millimeter of mercury.

Abbreviations: bpm = beats per minute, DBP = diastolic blood pressure, FFM = fat‐free mass, PP = pulse pressure, RPP = rate‐pressure product, SBP = systolic blood pressure.

### 3.2. Pulmonary Function

The Brunner–Langer analysis showed no significant Group × Time interaction for FVC (ATS = 0.066, *p* = 0.798), FEV1 (ATS = 0.010, *p* = 0.922), FEV1/FVC (ATS = 0.060, *p* = 0.806), FEF25%–75% (ATS = 2.855, *p* = 0.091), or PEF (ATS = 1.998, *p* = 0.158). A significant main effect of Time was observed for PEF (ATS = 9.999, *p* = 0.002), indicating an overall increase in PEF over time, without evidence of a differential intervention effect (Table 3).

**TABLE 3:** Pulmonary function parameters before and after the intervention in the control and RT groups.

Parameter	Time	Control group mean ± SD	95% CI	RT group mean ± SD	95% CI	Group × Time ATS	*p*‐value
FVC (%)	Pre	95.94 ± 15.61	88.18–103.71	93.85 ± 19.01	86.33–101.37	0.066	0.798
	Post	98.00 ± 16.80	89.65–106.35	92.96 ± 18.88	85.49–100.43		

FEV_1_ (%)	Pre	92.56 ± 15.56	84.82–100.30	91.41 ± 21.54	82.89–99.93	0.010	0.922
	Post	97.11 ± 15.00	89.65–104.57	91.93 ± 20.38	83.86–99.99		

FEV_1_/FVC (%)	Pre	96.33 ± 5.14	93.77–98.89	97.19 ± 9.70	93.35–101.02	0.060	0.806
	Post	99.11 ± 4.80	96.72–101.50	98.78 ± 9.47	95.03–102.52		

FEF_25_–_75_ (%)	Pre	87.28 ± 21.05	76.81–97.75	92.96 ± 37.49	78.13–107.79	2.855	0.091
	Post	97.50 ± 24.57	85.28–109.72	91.67 ± 32.48	78.82–104.51		

PEF (%)	Pre	61.39 ± 17.69	52.59–70.19	64.37 ± 20.62	56.22–72.53	1.998	0.158
	Post	72.72 ± 18.18	63.68–81.76	67.48 ± 17.64	60.50–74.46		

*Note:* Data are expressed as mean ± standard deviation (SD) and 95% confidence interval (95% CI). FEV_1_ = forced expiratory volume in one second; FEV_1_/FVC = ratio of forced expiratory volume in one second to forced vital capacity; FEF_25_–_75_ = forced expiratory flow at 25%–75% of the pulmonary volume. Group × Time effects were evaluated using the Brunner–Langer approach for factorial longitudinal data and are reported as ANOVA‐type statistics (ATS) with corresponding *p*‐values.

Abbreviations: FVC = forced vital capacity, PEF = peak expiratory flow.

The bootstrap analysis showed a between‐group difference in change of +1.23% points for FVC (95% CI −3.43 to 5.51), +0.58% points for FEV1 (95% CI −4.87 to 5.85), −0.51% points for FEV1/FVC (95% CI −3.49 to 2.62), +7.96% points for FEF25%–75% (95% CI −3.27 to 19.21), and +7.90% points for PEF (95% CI 0.28–16.09).

In adjusted sensitivity analyses accounting for the corresponding baseline value, hypertension, hypercholesterolemia, and smoking history, no significant between‐group differences were observed for FVC (*p* = 0.993), FEV1 (*p* = 0.934), FEV1/FVC (*p* = 0.962), FEF25%–75% (*p* = 0.600), or PEF (*p* = 0.175). For FEF25%–75%, which showed evidence of heteroscedasticity, a generalized linear model with robust covariance estimates was used; adjusted postintervention means were 94.90 (95% CI 85.04–104.76) for CON and 98.23 (95% CI 89.06–107.39) for RT, with a between‐group difference of −3.33 (95% CI −15.75 to 9.10; *p* = 0.600).

### 3.3. Respiratory Muscle Strength, Functional Performance, Oxygen Saturation, and Handgrip Strength

Significant Group × Time interactions were observed for MIP (ATS = 9.926, *p* = 0.002) and MEP (ATS = 8.111, *p* = 0.004). For MIP, the within‐group analysis showed a significant pre‐to‐post change in the RT group (*p* < 0.001), but not in the CON group (*p* = 1.000), indicating an improvement in inspiratory muscle strength following the intervention. Between‐group differences were not significant at baseline (*p* = 1.000) but were significant postintervention (*p* = 0.004) (Figure 2A). For MEP, a significant pre‐to‐post change was observed in the RT group (*p* = 0.002), but not in the CON group (*p* = 0.491); the groups differed significantly postintervention (*p* = 0.001), but not at baseline (*p* = 0.491) (Figure 2B). The bootstrap analysis estimated a between‐group difference in change of −18.61 cmH_2_O (95% CI −30.22 to −7.14) for MIP and +14.87 cmH_2_O (95% CI 3.46–26.10) for MEP.

**FIGURE 2:**
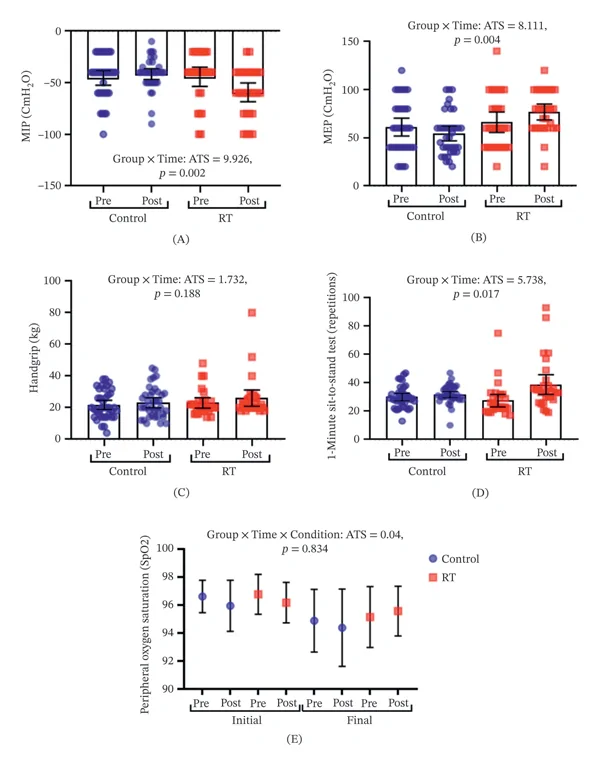
Effects of RT on respiratory muscle strength, functional performance, and oxygen saturation. Effects of resistance training on respiratory muscle strength, functional performance, and oxygen saturation (SpO_2_). (A) Maximal inspiratory pressure (MIP, cmH_2_O). (B) Maximal expiratory pressure (MEP, cmH_2_O). (C) Handgrip (Kg). (D) 1‐minute sit‐to‐stand test (repetitions). (E) Peripheral oxygen saturation (SpO_2_, %). Outcomes were assessed in the control (CON) and resistance training (RT) groups before (Pre) and after (Post) the intervention. Individual observations are shown as points; horizontal lines and error bars represent the group mean and 95% confidence interval (CI), respectively. ANOVA‐type statistics (ATS) and corresponding p‐values for the Group × Time effect are shown in the respective panels. CON, control group (*n* = 38); RT, resistance training group (*n* = 31); Pre, pre‐intervention; Post, postintervention. Statistical significance was set at *p* < 0.05.

Handgrip strength also showed a significant main effect of Time (ATS = 9.520, *p* = 0.002), but neither the Group × Time interaction (ATS = 1.732, *p* = 0.188) nor the Group effect (ATS = 0.416, *p* = 0.519) was significant. The bootstrap estimate of the between‐group difference in change was +0.29 kg (95% CI −1.58 to 2.14) (Figure 2C).

For the 1‐MSTST, significant main effects of Time (ATS = 22.508, *p* < 0.001) and Group × Time interaction (ATS = 5.738, *p* = 0.017) were observed. The RT group showed a significant pre‐to‐post improvement (*p* < 0.001), whereas the change in the CON group was not significant (*p* = 0.145). No significant between‐group differences were observed at baseline (*p* = 0.145) or postintervention (*p* = 0.227). The bootstrap estimate of the between‐group difference in change was +9.58 repetitions (95% CI 3.64–16.04) (Figure 2D).

For SpO_2_, the Brunner–Langer analysis showed a significant main effect of Time (ATS = 46.81, df = 1, *p* < 0.001), whereas the Group × Time × Condition interaction was not significant (ATS = 0.04, df = 1, *p* = 0.834), indicating no differential pre‐to‐post SpO_2_ response between the RT and control groups over time. The between‐group difference in change was 0.11% points (95% CI −2.05 to 2.37) (Figure 2E).

In adjusted sensitivity analyses, significant between‐group differences remained for MIP (*p* = 0.012) and MEP (*p* = 0.005) after accounting for the corresponding baseline outcome, hypertension, hypercholesterolemia, and smoking history. For MIP, robust covariance estimates were used because of heteroscedasticity; adjusted means were −41.52 (95% CI −50.50 to −32.53) for CON and −54.07 (95% CI −64.65 to −43.48) for RT. For MEP, adjusted means were 56.81 (95% CI 48.48–65.14) and 73.01 (95% CI 64.02–82.01), respectively. For the 1‐MSTST, the adjusted between‐group comparison did not reach statistical significance (*p* = 0.100), despite the significant Group × Time interaction in the primary longitudinal analysis.

### 3.4. Plasma Biomarkers

Significant Group × Time interactions were observed for plasma IL‐6 (ATS = 32.62, *p* < 0.001), IL‐10 (ATS = 32.94, *p* < 0.001), and Klotho (ATS = 98.55, *p* < 0.001). For IL‐6, the RT group showed a significant pre‐to‐post reduction (*p* < 0.001), whereas the CON group did not show a significant change (*p* = 0.758). The groups did not differ significantly at baseline (*p* = 0.104), but differed significantly postintervention (*p* < 0.001) (Figure 3A). For IL‐10, a significant pre‐to‐post increase was observed in the RT group (*p* < 0.001), but not in the CON group (*p* = 0.226); the groups differed significantly at baseline (*p* = 0.031) and postintervention (*p* < 0.001) (Figure 3B). For Klotho, significant pre‐to‐post changes were observed in both the CON (*p* < 0.001) and RT (*p* = 0.002) groups, with significant between‐group differences at baseline (*p* = 0.002) and postintervention (*p* < 0.001) (Figure 3C).

FIGURE 3Effects of resistance training on systemic and airway inflammatory mediators and Klotho levels. Plasma and exhaled breath condensate (EBC) biomarkers were measured in the control (CON) and resistance training (RT) groups before (Pre) and after (Post) the intervention. (A) Plasma IL‐6 (pg/mL). (B) Plasma IL‐10 (pg/mL). (C) Plasma Klotho (pg/mL). (D) Plasma TNF‐α (pg/mL). (E) EBC IL‐6 (pg/mL). (F) EBC IL‐10 (pg/mL). (G) EBC Klotho (pg/mL). (H) EBC TNF‐α (pg/mL). Individual observations are shown as points; horizontal lines and error bars represent the group mean and 95% confidence interval (CI), respectively. ANOVA‐type statistics (ATS) and corresponding p‐values for the Group × Time effect are shown in the respective panels. CON, control group (*n* = 38); RT, resistance training group (*n* = 31); Pre, pre‐intervention; Post, postintervention; EBC, exhaled breath condensate. Statistical significance was set at *p* < 0.05.

**Figure:**
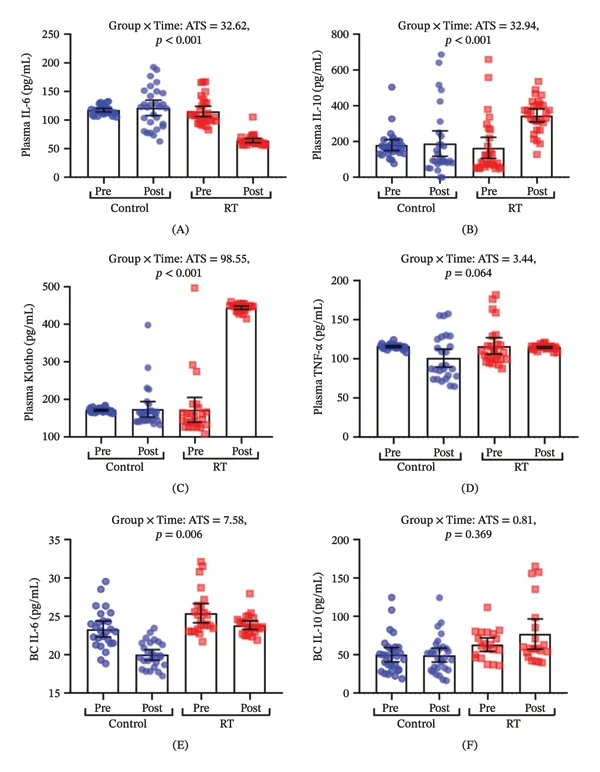

**Figure:**
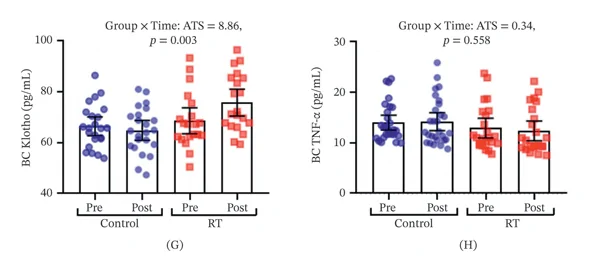

The bootstrap estimates of the between‐group difference in change were +55.10 for plasma IL‐6 (95% CI 40.10–70.69), −172.18 for plasma IL‐10 (95% CI −262.89 to −73.23), and −269.29 for plasma Klotho (95% CI −302.13 to −228.20). Plasma TNF‐α showed no significant Group × Time interaction (ATS = 3.44, *p* = 0.064) and no significant main effect of Time (ATS = 2.56, *p* = 0.110). The bootstrap estimate of the between‐group difference in change was +19.35 (95% CI −7.99 to 50.69).

Adjusted sensitivity analyses showed that the between‐group differences remained significant for plasma IL‐6 (*p* < 0.001), IL‐10 (*p* = 0.008), and Klotho (*p* < 0.001) after accounting for baseline biomarker values, hypertension, hypercholesterolemia, and smoking history. Robust covariance estimates were used for IL‐6, IL‐10, and Klotho because of heteroscedasticity. Adjusted postintervention means were 78.16 (95% CI 71.72–84.60) for CON and 62.30 (95% CI 57.99–66.60) for RT for IL‐6; 103.63 (95% CI 72.10–135.15) and 47.83 (95% CI 26.57–69.10) for IL‐10, respectively; and 252.71 (95% CI 205.20–300.22) and 449.47 (95% CI 425.89–473.06) for Klotho, respectively. In contrast, no adjusted between‐group difference was observed for plasma TNF‐α (*p* = 0.844). Because baseline Klotho concentrations differed between groups, baseline Klotho was included as a covariate in the adjusted model. The group effect remained significant after adjustment (*p* < 0.001).

### 3.5. EBC Biomarkers

Significant Group × Time interactions were observed for EBC IL‐6 (ATS = 7.58, *p* = 0.006) and EBC Klotho (ATS = 8.86, *p* = 0.003). For EBC IL‐6, significant pre‐to‐post reductions were observed in both the CON (*p* = 0.014) and RT (*p* < 0.001) groups. The groups did not differ significantly at baseline (*p* = 0.051), but differed significantly postintervention (*p* < 0.001) (Figure 3E). For EBC Klotho, a significant pre‐to‐post increase was observed in the RT group (*p* < 0.001), whereas the CON group did not show a significant change (*p* = 0.573). The groups did not differ significantly at baseline (*p* = 0.741), but differed significantly postintervention (*p* = 0.004) (Figure 3G).

The bootstrap estimates of the between‐group difference in change were −0.21 for EBC IL‐6 (95% CI −2.44 to 2.39) and −12.30 for EBC Klotho (95% CI −20.26 to −4.87). No significant Group × Time interactions were observed for EBC IL‐10 (ATS = 0.81, *p* = 0.369) or EBC TNF‐α (ATS = 0.34, *p* = 0.558). The bootstrap estimates of the between‐group difference in change were −57.90 for EBC IL‐10 (95% CI −115.65 to −9.35) and +0.73 for EBC TNF‐α (95% CI −1.14 to 2.74) and were considered complementary to the primary longitudinal interaction analyses (Figure 3F,H).

In adjusted sensitivity analyses accounting for the corresponding baseline biomarker value, hypertension, hypercholesterolemia, and smoking history, no significant between‐group differences were observed for EBC IL‐6 (*p* = 0.477), TNF‐α (*p* = 0.844), or Klotho (*p* = 0.991). For EBC IL‐10, a generalized linear model with robust covariance estimates was used because of heteroscedasticity. The adjusted postintervention means were 56.19 (95% CI 41.80–70.57) for CON and 70.39 (95% CI 59.61–81.17) for RT, with a between‐group difference of −14.20 (95% CI −28.16 to −0.24; *p* = 0.046).

To account for the potential increase in type I error associated with the multiple biomarker comparisons, a Holm‐adjusted sensitivity analysis was performed across the eight Group × Time comparisons of plasma and EBC inflammatory and fibrotic biomarkers. The significant interactions remained statistically significant for plasma IL‐6 (adjusted *p* = 0.008), plasma IL‐10 (adjusted *p* = 0.008), plasma Klotho (adjusted *p* = 0.008), EBC IL‐6 (adjusted *p* = 0.024), and EBC Klotho (adjusted *p* = 0.015). The Group × Time interactions remained nonsignificant for plasma TNF‐α (adjusted *p* = 0.192), EBC IL‐10 (adjusted *p* = 0.738), and EBC TNF‐α (adjusted *p* = 0.738). For comparisons originally reported as *p* < 0.001, a value of 0.001 was used for the multiplicity‐adjustment calculation.

### 3.6. Sensitivity Analyses

Sensitivity analyses largely supported the primary intervention‐related findings. For SpO_2_, the Group × Time × Condition interaction remained nonsignificant after exclusion of five prespecified outlying observations (ATS = 0.04, df = 1, *p* = 0.833), compared with the primary analysis including all observations (ATS = 0.04, df = 1, *p* = 0.834). Although the Time × Condition interaction changed from significant in the primary analysis (ATS = 4.99, df = 1, *p* = 0.026) to nonsignificant after exclusion of the outlying observations (ATS = 3.81, df = 1, *p* = 0.051), this did not affect the interpretation regarding the intervention, as the three‐way Group × Time × Condition interaction remained consistently nonsignificant. For plasma Klotho, the Group × Time interaction remained significant after exclusion of the four prespecified extreme observations (ATS = 152.607, *p* < 0.001), compared with the primary analysis including all observations (ATS = 98.55, *p* < 0.001).

Overall, the adjusted analyses further supported the between‐group effects observed for MIP, MEP, and selected plasma biomarkers, whereas the between‐group comparison for the 1‐MSTST was attenuated after adjustment.

## 4. Discussion

This randomized controlled trial examined the effects of a 12‐week RT program on lung function, respiratory muscle strength, functional performance, and systemic and airway inflammatory biomarkers in older adults without recent participation in structured exercise programs. RT significantly improved inspiratory and expiratory respiratory muscle strength and was associated with differential responses in selected systemic and airway biomarkers, while evidence for an effect on functional performance was less consistent. However, such results are not dependent on each other and a causal relationship was not tested. The biomarker findings should be interpreted as intervention‐associated biological responses rather than as definitive evidence of immunomodulation or specific anti‐inflammatory mechanisms. Our findings support previous evidence suggesting that RT may improve respiratory muscle strength in older adults and provide preliminary evidence of a differential longitudinal response in functional performance. Importantly, this appears to be the first study to demonstrate changes in EBC biomarkers following a 12‐week RT intervention in this population, providing preliminary insight into potential local airway responses to RT. Although only a selected set of biomarkers was measured (IL‐6, TNF‐α, IL‐10, and Klotho), these mediators are involved in pro‐ and anti‐inflammatory pathways and may provide insight into biological patterns associated with RT. Together, these findings contribute to the current understanding of how RT may promote respiratory muscle adaptations and may be associated with selected functional and biological responses during aging.

Impaired lung function in older adults is a known predictor of morbidity and mortality [54]. Age‐related structural changes, such as increased alveolar diameter and reduced elastic recoil, may contribute to gradual declines in respiratory performance [54]. In addition, aging is commonly associated with reductions in respiratory muscle strength and chest‐wall compliance, which may negatively affect respiratory performance and functional exercise capacity [55, 56]. Consequently, lung function decline is often reflected in reductions in FEV1 and FVC [45, 56, 57].

In the present study, no significant changes were observed in FVC%, FEV1%, or the FEV1/FVC ratio in either the RT or control groups. These findings are consistent with previous studies in older adults, including investigations of high‐intensity RT, which reported maintenance of spirometry parameters without measurable improvements [57, 58]. The lack of effect on pulmonary function may be explained physiologically by the fact that RT primarily targets skeletal muscles and does not directly modify lung parenchyma or airway mechanics; therefore, standard spirometric measures may be insensitive to the muscular adaptations induced by RT in older adults [59].

Importantly, the absence of significant changes in spirometry parameters does not necessarily indicate an absence of respiratory adaptations following RT. Spirometry primarily assesses lung volumes and airflow, whereas RT may promote neuromuscular adaptations that influence respiratory muscle performance [60, 61]. Accordingly, the significant improvements in MIP and MEP observed in the present study may reflect enhanced force‐generating capacity of the inspiratory and expiratory muscles rather than structural changes in lung tissue or airway caliber. This distinction may explain the observed improvements in respiratory muscle strength in the absence of significant changes in conventional pulmonary function indices.

These findings are consistent with previous studies reporting beneficial effects of RT on respiratory muscle performance in older adults [61–63]. Although the underlying physiological mechanisms were not directly assessed, the observed changes may be related to neuromuscular adaptations, improved motor‐unit recruitment, and enhanced force production during maximal respiratory maneuvers. From a functional perspective, greater respiratory muscle strength may contribute to ventilatory support during physical activities; however, the present study did not directly assess ventilatory reserve, exercise tolerance, or respiratory muscle endurance. Therefore, these potential functional implications should be interpreted as physiological considerations rather than direct outcomes demonstrated by the study.

From a clinical perspective, the observed improvements in MIP and MEP are relevant because respiratory muscle strength is associated with respiratory performance and functional health in older adults. Nevertheless, universally accepted minimal clinically important differences for these measures in community‐dwelling older adults have not been established. Thus, although the statistically significant changes support a beneficial effect of RT on respiratory muscle strength, the extent to which these changes translate into clinically meaningful improvements in daily functioning requires further investigation [64–66].

Handgrip strength increased significantly from pre‐ to postintervention in both groups. Although the RT group showed a numerically greater improvement, the absence of a significant Group × Time interaction indicates that the change did not differ significantly between groups. Therefore, the findings do not establish a specific effect of RT on handgrip strength compared with the control condition. The improvement observed in both groups should be interpreted in light of the study design and the possibility of factors unrelated to the intervention contributing to changes over time [66–68].

Functional capacity, assessed using the 1‐MSTST, demonstrated a differential longitudinal response between groups, with a significant improvement following RT but not in the control group. However, the adjusted postintervention comparison was not statistically significant after accounting for baseline sit‐to‐stand performance, hypertension, hypercholesterolemia, and smoking history. Therefore, the findings provide evidence of a differential longitudinal response but should be interpreted cautiously as evidence of a between‐group intervention effect. This finding is consistent with previous evidence that RT can improve lower‐limb strength, muscular endurance, and performance in functional tasks among older adults [69, 70]. The improvement observed in the RT group may be related to neuromuscular adaptations, enhanced motor‐unit recruitment, and improved force production during repeated sit‐to‐stand movements. However, because the study did not directly assess the specific mechanisms underlying this improvement, these explanations remain interpretative.

The present findings indicate that RT may produce adaptations across different physiological domains, including respiratory muscle strength and functional performance. In contrast, spirometry parameters did not demonstrate significant Group × Time interactions, suggesting that the effects of the intervention were not uniform across all respiratory outcomes. The observed changes in systemic and airway biomarkers further suggest that RT was associated with differential responses in selected inflammatory and aging‐related biomarkers. However, the clinical significance of these biomarker changes remains uncertain because validated minimal clinically important differences have not been established for IL‐6, IL‐10, TNF‐α, or Klotho concentrations in older adults. Consequently, these findings should be interpreted as evidence of physiological changes rather than definitive evidence of clinical benefit.

Analysis of oxygen saturation during the 1‐MSTST showed no meaningful difference in the SpO_2_ response between the RT and control groups over the intervention period. Oxygen saturation remained relatively stable during the test, with only small variations and values maintained above 94%, suggesting preserved oxygenation during this brief functional challenge. These small variations are unlikely to be clinically relevant [71], as SpO_2_ remained within normal physiological ranges throughout the test. The absence of a distinct intervention‐related effect on oxygen saturation suggests that the functional response to RT was not accompanied by measurable changes in peripheral oxygen saturation during this short‐duration exercise test [72]. Overall, these findings indicate that oxygen saturation was largely preserved during the 1‐MSTST, with limited clinical relevance of the observed variations.

Research shows that frail older adults can develop low‐grade chronic inflammation even in the absence of infection [73, 74]. This persistent inflammation is linked to increased morbidity and mortality and interacts bidirectionally with age‐related conditions, such as cardiovascular disease, neurodegeneration, cancer, and frailty [75, 76]. In the present study, baseline serum concentrations of IL‐6, TNF‐α, IL‐10, and Klotho were within ranges typically observed in healthy older adults, consistent with previous reports [76–80], indicating that participants did not exhibit markedly elevated inflammatory biomarker concentrations at baseline. However, baseline concentrations were not entirely balanced between groups, with significant between‐group differences observed for plasma IL‐10 and Klotho. These baseline differences were considered when interpreting the longitudinal and postintervention findings.

Following the 12‐week RT protocol, significant between‐group differences were observed in changes in plasma IL‐6, IL‐10, and Klotho, whereas no significant between‐group difference was observed for plasma TNF‐α. These findings align with previous studies showing that RT can be associated with changes in systemic inflammatory biomarkers in older adults; potential mechanisms proposed in previous literature include reduced visceral adiposity, enhanced myokine release, improved redox balance, and modulation of immune function [80–82]. To our knowledge, however, few studies have investigated the effects of RT on circulating Klotho concentrations in older adults without participation for at least 1 year in structured exercise programs. The present study further provides preliminary evidence that RT was associated with changes in circulating Klotho concentrations. Although the observed changes are consistent with a potential alteration in inflammatory and aging‐related biological signaling, clinically meaningful thresholds for IL‐6, IL‐10, TNF‐α, and Klotho have not yet been established in older adults, particularly for measurements obtained from EBC. Therefore, these findings should be interpreted as evidence of biological responses associated with the intervention rather than established clinical benefit.

The baseline imbalance in plasma Klotho warrants particular consideration. Baseline concentrations also differed significantly between groups for plasma IL‐10. These baseline differences were considered in the interpretation of the postintervention findings and were addressed in the adjusted sensitivity analyses. Because baseline Klotho concentrations differed between groups, baseline Klotho was included as a covariate together with hypertension, hypercholesterolemia, and smoking history in the adjusted sensitivity analysis. The between‐group difference in postintervention Klotho remained statistically significant after adjustment (*p* < 0.001), whereas baseline Klotho was not statistically significant in the adjusted model (*p* = 0.068). The persistence of the group effect after adjustment indicates that the baseline imbalance alone did not account for the observed postintervention difference. Nevertheless, the presence of a baseline difference limits the extent to which the Klotho findings can be interpreted as an unambiguous intervention effect and therefore warrants cautious interpretation.

Potential mechanisms that may contribute to the observed biomarker responses include reductions in visceral adiposity, repeated transient increases in anti‐inflammatory myokines released during exercise, improved oxidative balance, and attenuation of chronic low‐grade inflammation commonly associated with aging [83, 84]. In addition, RT may influence pulmonary immune responses through changes in systemic inflammatory signaling and circulating mediators [28]. However, these mechanisms were not directly measured in the present study and therefore remain hypothetical explanations for the observed biomarker patterns.

To our knowledge, this is the first study to investigate inflammatory biomarkers in EBC following a RT program in older adults. In the present study, RT was associated with differential changes in airway IL‐6 and Klotho, whereas no significant between‐group differences were observed for IL‐10 or TNF‐α. Importantly, significant pre‐to‐post reductions in EBC IL‐6 were observed in both the CON and RT groups. Although the Group × Time interaction was significant, the adjusted postintervention comparison was not statistically significant (*p* = 0.477). Therefore, this finding should not be interpreted as evidence of an intervention‐specific reduction in airway inflammation. Rather, it represents a differential longitudinal biomarker response that should be interpreted cautiously. Notably, the pattern of airway biomarker changes showed some similarities to the systemic findings, particularly for IL‐6 and Klotho. These findings provide preliminary evidence that RT was associated with changes in selected systemic and airway inflammatory and aging‐related biomarkers in older adults and are consistent with previous reports demonstrating exercise‐related modulation of inflammatory pathways in this population [49].

However, because only a limited panel of biomarkers was evaluated and measurements were obtained from EBC, these findings should not be interpreted as evidence of a generalized reduction in pulmonary inflammation. The observed airway biomarker responses may reflect exercise‐related changes in systemic inflammatory signaling or indirect effects on airway inflammatory homeostasis; however, these mechanisms were not directly assessed in the present study. Moreover, because EBC IL‐6 also decreased in the control group and the adjusted postintervention comparison was not statistically significant, the observed changes cannot be attributed specifically to RT.

Because multiple biomarker comparisons were performed, the possibility of type I error was also considered. The significant Group × Time interactions for plasma IL‐6, IL‐10, Klotho, and EBC IL‐6 and Klotho remained significant after Holm adjustment, indicating that these findings were not eliminated by the multiplicity correction. Nevertheless, statistical significance alone does not establish immunomodulation, attenuation of airway inflammation, or a specific causal biological mechanism.

### 4.1. Limitations

An additional consideration is the unequal distribution of cardiometabolic comorbidities between groups. Diabetes mellitus was present in 7/38 (18.4%) CON and 4/31 (12.9%) RT participants, hypercholesterolemia in 11/38 (28.9%) and 4/31 (12.9%), and systemic hypertension in 27/38 (71.1%) and 14/31 (45.2%), respectively. No participant was a current smoker, whereas former smoking was reported in 10/38 (26.3%) CON and 4/31 (12.9%) RT participants. Pharmacological treatment was homogeneous within each comorbidity: Participants with systemic hypertension were treated with losartan, those with type 2 diabetes mellitus with metformin, and those with hypercholesterolemia with simvastatin. These treatments remained unchanged during the intervention period.

Although the uniformity of medication type within each comorbidity reduces pharmacological heterogeneity, the higher prevalence of cardiometabolic diseases in the CON group also resulted in a greater proportion of participants exposed to these medications. This may be relevant because losartan may modulate renin–angiotensin signaling, oxidative stress, and inflammatory pathways; metformin has metabolic and anti‐inflammatory effects; and simvastatin exerts pleiotropic anti‐inflammatory and endothelial effects in addition to lowering cholesterol. Moreover, hypertension, type 2 diabetes, and hypercholesterolemia themselves are associated with alterations in systemic inflammation, oxidative stress, and endothelial function. Therefore, both differences in underlying cardiometabolic disease burden and corresponding medication exposure may have contributed to variability in the inflammatory and Klotho responses observed in this study.

This consideration is particularly relevant to the significant Group × Time interactions observed for plasma IL‐6, IL‐10, and Klotho and EBC IL‐6 and Klotho. To account for the potential influence of baseline clinical characteristics, adjusted sensitivity analyses included hypertension, hypercholesterolemia, smoking history, and the corresponding baseline outcome. These analyses provided additional evidence regarding the between‐group differences observed for MIP, MEP, and selected plasma biomarkers, whereas the adjusted comparison for sit‐to‐stand was no longer statistically significant. For EBC biomarkers, adjusted differences were not significant for IL‐6, TNF‐α, or Klotho; EBC IL‐10 showed a significant adjusted postintervention difference in a robust model. Because these adjusted analyses addressed postintervention between‐group differences rather than longitudinal Group × Time interactions, they were interpreted as complementary sensitivity analyses rather than replacements for the primary longitudinal analysis.

For EBC IL‐10 specifically, the primary longitudinal analysis did not demonstrate a significant Group × Time interaction, whereas the adjusted postintervention comparison was statistically significant. Because these analyses address different statistical questions, the adjusted finding should be considered complementary rather than evidence of a significant longitudinal intervention effect. In addition, the biomarker panel was limited to IL‐6, TNF‐α, IL‐10, and Klotho and may not capture the full complexity of systemic and pulmonary inflammatory responses.

Respiratory muscle strength was evaluated using absolute MIP and MEP values rather than predicted values adjusted for age and sex, and respiratory mechanics, ventilatory efficiency, and respiratory muscle activation were not directly assessed. Therefore, mechanistic interpretations of the observed respiratory adaptations should remain cautious. Overall, spirometric values were within the expected range, with mean FVC and FEV1 values close to predicted values and preserved FEV1/FVC ratios, indicating generally preserved pulmonary function in the study population. Consequently, the absence of significant Group × Time interactions for most conventional spirometry parameters should not be interpreted as evidence of improvement in established ventilatory impairment. Finally, the 1‐min sit‐to‐stand findings should be interpreted cautiously because, although a significant Group × Time interaction and a significant within‐group improvement were observed in the RT group, the adjusted postintervention between‐group comparison was not statistically significant (*p* = 0.100) [85–87].

## 5. Conclusion

In conclusion, 12 weeks of supervised RT promoted significant adaptations in respiratory muscle strength, functional capacity, and selected systemic and airway biomarkers in older adults. The significant Group × Time interactions for MIP and MEP, the 1‐MSTST, and markers related to inflammation and cellular aging indicate that RT may influence multiple physiological pathways associated with healthy aging. These adaptations occurred without significant between‐group changes in pulmonary function, suggesting that the benefits of RT may be more evident in respiratory muscle performance, functional capacity, and inflammatory and aging‐related regulation than in conventional spirometric parameters. Overall, the findings support supervised RT as a relevant nonpharmacological intervention for promoting functional and physiological health in older adults.

## Author Contributions

The authorship followed the International Committee of Medical Journal Editors (ICMJE). Anamei Silva‐Reis: investigation, data curation, formal analysis, and drafting of the manuscript. Rômulo Antonio Costa de Oliveira, Meiry Souza Moura‐Maia, Eduarda Teodora Rachid‐Wolpp, Naiara Nadia Moreira‐Silva, and Luis Vicente Franco de Oliveira: investigation, data curation, formal analysis, and critical revision of the article. Andre Luis Lacerda Bachi, Vicente Aprigliano, Omid Salehian, Regiane Albertini, Guilherme Eustáquio Furtado, and Daiana Stolz: data curation, formal analysis, and critical revision of the article. Alberto Souza Sá Filho: study concept and design, methodology, formal analysis, investigation, drafting of the manuscript, and critical revision of the article. Rodolfo P. Vieira: study concept and design, methodology, formal analysis, project administration, supervision, funding acquisition, drafting of the manuscript, and critical revision of the article.

## Funding

This research was funded by the Sao Paulo Research Foundation (FAPESP) for the research grant #2012/15165‐2 provided to Rodolfo P. Vieira. Rodolfo P. Vieira also thanks the Conselho Nacional de Desenvolvimento Científico e Tecnológico (CNPq) for the grant #314434/2026‐7. Anamei Silva‐Reis holds a PhD fellowship (#202310267000648) from Goias Research Foundation (FAPEG) and an Excellence PhD fellowship from FAPEG (#202610267000938). Eduarda Teodora Rachid‐Wolpp and Naiara Nadia Moreira‐Silva hold a scientific initiation fellowship from Evangelical Education Association (AEE).

## Disclosure

The funders had no role in the design of the study; collection, analysis, or interpretation of the data; writing of the manuscript; or decision to submit the manuscript for publication. All authors gave the final approval of the version to be published.

## Ethics Statement

Ethical approval was granted by the Evangelical University of Goiás Ethics Committee (protocol 6.095.284) on June 01, 2023.

## Consent

All volunteers provided verbal and written informed consent.

## Conflicts of Interest

The authors declare no conflicts of interest.

## Data Availability

The data supporting the findings of this study are available from the corresponding author upon request.
